# Hemophagocytic lymphohistiocytosis: contribution from clinical and laboratory criteria for the diagnosis

**DOI:** 10.1186/1939-4551-8-S1-A56

**Published:** 2015-04-08

**Authors:** Paula Campos Perim, Stella Arruda Miranda, Cristina Jacob, Ana Paula Moschione Castro, Antonio Carlos Pastorino, Mayra Dorna

**Affiliations:** 1 Unit of Allergy and Immunology, Brazil

## Background

To describe, among the diagnostic criteria proposed for Hemophagocytic Lymphohistiocytosis (HLH), which ones were the most valuable for the definition of the disease in pediatric patients in a Reference Center for Primary Immunodeficiencies (PID).

## Methods

It was a descriptive and retrospective study carried out in the period from 2009 to 2014, including patients diagnosed as familial or secondary HLH, from a Brazilian Pediatric Reference Center for PIDs. The criteria used for diagnosis was from Histiocyte Society HLH-2004 Protocol. All data was collected from patients' records.

## Results

Eight patients (4 males) were evaluated, being diagnosed 2 mutations in the perforin gene (in 3 patients, including twins), 3 secondary to Chèdiak-Higashi syndrome, 1 associated to Epstein-Baar virus infection, 1 associated to Kawasaki syndrome, and another unknown cause. The median age at diagnosis was 29,5 months (from 2 months to 12 years). The median time necessary to confirm HLH was 21 days (from 15 to 42 days), and the most precocious ones were in patients with genetic mutations. Fever was the first symptom presented by all patients, the incidence of thrombocytopenia was also 100%; anemia, hypertriglyceridemia and increased ferritin were presented by 87%; hypofibrinogenemia by 75%; neutropenia and splenomegaly by 62%; hemophagocytosis in bone marrow by 37%. The most frequent criteria combination was fever, thrombocytopenia, anemia, increased ferritin and hypertriglyceridemia. Soluble CD25 and NK-cell activity weren’t avaiable at the diagnosis. By the time HLH was established, all patients were receiving antibiotics. Their outcomes were 2 deaths, 2 bone marrow transplants with good evolution and 4 patients are still in follow-up.

## Conclusions

This study points out the importance to think about HLH as differential diagnosis, applying the diagnostic criteria, in order to proper diagnosis and treatment. It also highlights that hemophagocytosis in bone marrow, despite being characteristic, wasn’t found in all patients, proving that it isn’t essential to the diagnosis.

